# Enhancing scientific discoveries in molecular biology with deep generative models

**DOI:** 10.15252/msb.20199198

**Published:** 2020-09-25

**Authors:** Romain Lopez, Adam Gayoso, Nir Yosef

**Affiliations:** ^1^ Department of Electrical Engineering and Computer Sciences University of California Berkeley CA USA; ^2^ Center for Computational Biology University of California Berkeley CA USA; ^3^ Chan‐Zuckerberg Biohub San Francisco CA USA; ^4^ Ragon Institute of MGH, MIT, and Harvard Cambridge MA USA

**Keywords:** deep generative models, molecular biology, neural networks, Chromatin, Epigenetics, Genomics & Functional Genomics, Computational Biology

## Abstract

Generative models provide a well‐established statistical framework for evaluating uncertainty and deriving conclusions from large data sets especially in the presence of noise, sparsity, and bias. Initially developed for computer vision and natural language processing, these models have been shown to effectively summarize the complexity that underlies many types of data and enable a range of applications including supervised learning tasks, such as assigning labels to images; unsupervised learning tasks, such as dimensionality reduction; and out‐of‐sample generation, such as *de novo* image synthesis. With this early success, the power of generative models is now being increasingly leveraged in molecular biology, with applications ranging from designing new molecules with properties of interest to identifying deleterious mutations in our genomes and to dissecting transcriptional variability between single cells. In this review, we provide a brief overview of the technical notions behind generative models and their implementation with deep learning techniques. We then describe several different ways in which these models can be utilized in practice, using several recent applications in molecular biology as examples.

## Graphical abstract

This Review provides an introduction into deep generative models and their implementation with deep learning techniques. Several applications in genomics and single‐cell biology are presented, and the interpretability of such neural network models is discussed.

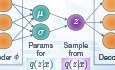

## Introduction

The widespread use of information‐rich technologies in the life sciences has spurred a lively crosstalk with research in machine learning, with the goal of improving how we explore and derive conclusions from biological data.

The most prevalent use of machine learning in this setting is *supervised learning*, where one makes use of any available observations in order to make inferences about measurable, yet unobserved quantities of interest. Consider as an example the case of a clinical study where one is interested to predict the outcome (discrete or quantitative) of treatments based on input covariates of environmental, genetic, or other molecular origins (Dincer *et al*, 2018; Rajkomar *et al*, 2018). In supervised learning, the input covariates (normally denoted by *x*) are usually available for all participants in the study, while the treatment outcome (i.e., the target, denoted by *y*) is only available for a subset of them. A classical approach to predict *y* in those missing cases is to learn a conditional distribution *p*(*y*|*x*)—namely what is the probability for a certain outcome *y* given any environmental or molecular information represented *by x*. Such an approach is referred to as *discriminative learning* and includes methods such as random forests, support vector machines, linear models, and neural networks (Box 1); we refer the reader to (Ching *et al*, 2018; Wainberg *et al*, 2018; Eraslan *et al*, 2019a; Zou *et al*, 2019) for reviews on the use of neural networks for supervised learning in biological settings.

While discriminative learning is a powerful approach, our focus in this review is on *generative* modeling—an alternative approach that explicitly models the joint distribution *p*(*x*,*y*). Naturally, generative modeling is attempting to solve a harder statistical problem, as it seeks to model the uncertainty in the input covariates *x* as well. Even though discriminative approaches generally have superior performance in the prediction problem, generative models are sometimes preferred as they can account for domain knowledge about how the data in *x* were generated (Ng & Jordan, 2002). For instance, if *x* represents an amino acid sequence, then the probability that *x* occurs in nature can be better estimated by a generative model that accounts for interactions between residues (Riesselman *et al*, 2018).

**Neural networks as function approximators**

A *neural network* describes a function *f* that composes simpler functions to learn complex mappings from input to output space. Neural networks are integral to deep generative models because they are theoretically capable of approximating any given function (Hornik *et al*, 1989), are efficient to train through backpropagation, and generalize well to unseen data through their inductive bias (preprint: Battaglia *et al*, 2018).

The neural network *architecture* specifies how the simpler functions are composed and how information flows through the network. A prevalent class of architectures is the so‐called feedforward network where computations can be viewed as chain of function compositions *f*(*x*) = *g* ^3^°*g* ^2^°*g* ^1^(*x*). Often these intermediate functions are non‐linear weighted sums (e.g., *g*(*x*) = *ψ*(∑_*i*_ *w*_*i*_ *x*_*i*_), where *ψ* represents a non‐linear *activation* function). Many problem‐specific architectures such as recurrent neural networks (RNN) or convolutional neural networks (CNN) have been developed and are reviewed in Zou *et al* (2019). Here, we highlight two widely used architectures in deep generative models: the multilayer perceptron and the autoencoder.

The multilayer perceptron (MLP) is one of the most common neural networks. It is characterized by an input layer, hidden layers (only one shown above with green nodes), and an output layer. The MLP is fully connected in the sense that the value of each node (circle in the figure) is a function of all the nodes in the previous layer (typically via the non‐linear weighted sum).

An autoencoder is defined by three modules: an encoder, bottleneck layer, and decoder. The encoder takes the input and reduces it to a lower dimension (through the bottleneck layer), and the decoder attempts to reconstruct the original input from the bottleneck. This architecture efficiently compresses the most salient information for reconstructing the data and serves as the backbone for the variational autoencoder.

Figure: Computational Schematics of the MLP and the autoencoder.

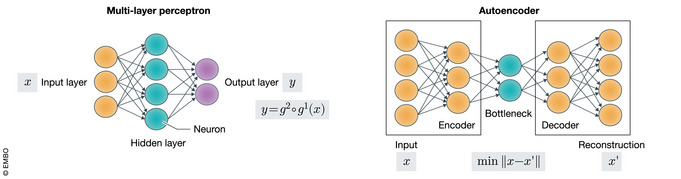

Explicitly modeling *x* means that generative models can also be used to generate new instances of *x in silico*. This capacity opens the way for additional applications that can aid with and even go beyond the prediction problem. Specifically, data generation can be used for identifying values of *x* that were not previously observed but are likely to be associated with a desirable value of *y*. This was used, for instance, to propose new chemical compounds (represented by *x*) that are likely to have a certain melting temperature (represented by *y*) (preprint: Sanchez‐Lengeling *et al*, 2017). Addition of new, artificial data points to an available set of observations has also been useful for increasing the performance of classifiers, e.g., in the context of predicting drug effects with fluorescence microscopy (Lafarge *et al*, 2019).

The use of generative models also extends beyond supervised learning, i.e., for cases where one does not have a target output to predict. In this *unsupervised* regime, we are interested in finding patterns in *x*, with common tasks including identifying measurements errors (outlier detection (Ding *et al*, 2018)), inferring the values of missing entries in *x* (imputation (Mattei & Frellsen, 2019)), or otherwise identifying latent sources of variation that give rise to the data (dimensionality reduction or embedding (Lopez *et al*, 2018a)).

Generative modeling has emerged as a powerful paradigm to address these tasks due to its ability to directly model *x*. For instance, in single‐cell RNA sequencing (where *x* corresponds to gene expression values in each cell), a common practice is to model *x* by conditioning on a small set of latent variables *z* that controls the generative process. Estimating *p*(*x*|*z*) provides a way to impute data entries that are missing due to low sensitivity. Conversely, estimating *p*(*z*|*x*) provides a way for embedding the cells in an informative low‐dimensional space (represented by *z*) that facilitates the clustering and identification of key latent sources of variation. In a different context, generative models were used to estimate the marginal likelihood *p*(*x*) of protein sequences (represented by *x*), providing a way to identify sequences that are not likely to emerge in evolution, and thus of decreased functionality (Riesselman *et al*, 2018).

Once the form of the generative model *p*(*x*,* z*) is posed, we aim to perform inference: fit the model to data. For example, a widely adopted paradigm is that of Bayesian inference, which consists of estimating the posterior distribution *p*(*z*|*x*). This posterior can then be utilized for many downstream tasks. Bayesian inference is often intractable due to the need to estimate the normalization constant of the distribution *p*(*x*). A recent review describes the main approximate inference methods (Markov chain Monte Carlo (Andrieu *et al*, 2003) and variational inference (Jordan *et al*, 1999)) as well as applications for high‐throughput data in biology (Yau & Campbell, 2019).

Our focus for this review is the recent developments of deep generative models (DGMs) and their applications in molecular biology. A DGM is a probabilistic framework that contains both a generative model and an inference procedure and in which either the model or the inference makes use of neural networks. In particular, we elaborate on recent applications of variational autoencoders (VAEs) (Kingma & Welling, 2014; Rezende *et al*, 2014) and generative adversarial networks (GANs) (Goodfellow *et al*, 2014). The VAE performs Bayesian inference using a variational approximation to the posterior *p*(*z*|*x*) parameterized with neural networks. The GAN performs a distinct type of inference (sometimes referred to as adversarial learning) and learns a simulator that mimics the data, also parameterized with neural networks. A DGM, like any other generative model, can be employed for making statistical inferences and reasoning about biologically meaningful hypotheses. The use of deep learning brings important pragmatic advantages: First, neural networks can be used as part of black‐box inference frameworks, which makes it especially easy for a practitioner to refine their modeling hypotheses without designing a whole new algorithm. For example, Grønbech *et al* (2020) report goodness of fit for 12 variants of a novel model for single‐cell RNA sequencing data, where each variant has a different generative model but the inference is mainly unchanged. Second, due to enormous progress on the neural network engineering side (Box 2), DGMs can scale up to large data sets, which have become common in the life sciences, such as transcriptome profiles of millions of single cells (Angerer *et al*, 2017), thousands of fluorescence microscopy images (Lafarge *et al*, 2019), or large collections of chemical compounds (preprint: Sanchez‐Lengeling *et al*, 2017; preprint: Guimaraes *et al*, 2018).

**Common implementations of neural networks**

The increasingly popular use of generative models in biomedical research is enabled and largely driven by extensive engineering work. The recent development of stable deep learning libraries such as Theano (preprint: The Theano Development Team, 2016), TensorFlow (preprint: Abadi *et al*, 2016), PyTorch (Paszke *et al*, 2019), and others saved the end‐user from investing in necessary infrastructure work, such as writing code for graphic processing units (GPUs) or deriving gradients of objective functions. A second crucial line of empirical work established some standard for neural network architectures and training procedures such as dropout (Srivastava *et al*, 2014), batch normalization (Ioffe & Szegedy, 2015), and non‐linearities (Glorot *et al*, 2011). These standards helped produce state‐of‐the‐art results on computer vision tasks and stabilized the training of neural network‐based model. A third line of work focuses on the important problem of automatic machine learning, which is the problem of architecture and hyperparameter search for neural networks (Kandasamy *et al*, 2018). Finally, as uncertainty quantification has become central in machine learning research, an important development paired neural networks with statistical inference via the popularization of probabilistic languages such as Stan (Carpenter *et al*, 2017), Pyro (Bingham *et al*, 2019), Edward (Tran *et al*, 2017a), and TensorFlow Probability (preprint: Dillon *et al*, 2017).

**Figure 1:**
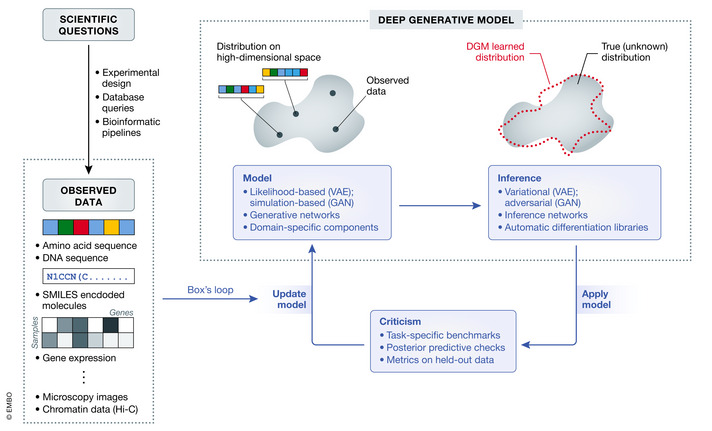
Overview of the modeling process with DGMs Research in molecular biology stems from the formulation of hypotheses. Such questions can be studied through the lens of a wide variety of data forms such as biological sequences, molecules, gene expression, or imaging data. The broad goal of a DGM is to estimate the distribution that generated the observed data. Constructing a DGM involves iterating through the steps of Box's loop. First, a model with domain‐specific components or assumptions is designed. Second, an inference procedure learns the optimal model parameters. Third, the model is criticized. This step consists of benchmarking the model on data sets and evaluating the goodness of fit of the model. Finally, the model is updated based on the criticism, starting a new iteration of the loop.

In the following, we provide a brief overview of the notions behind generative modeling and summarize several popular model types and their implementations (Fig 1). We then proceed to a more in‐depth description of applications in molecular biology. While our discussion will span a large set of case studies, we selected three leading examples to be used throughout the manuscript, which we present next.

### Leading examples

To illustrate the use of DGMs for modeling and investigating biological data, we focused on three specific case studies. We selected these cases as they cover a range of data types (single‐cell transcriptomics measurements, biological sequences, and three‐dimensional molecular structures), model types (VAE and GAN), and neural network architectures (fully connected, recurrent, and convolutional neural networks). These case studies also provide open‐source implementations.

The first selected model is single‐cell variational inference (scVI (Lopez *et al*, 2018a), Fig 2A), which aims to provide a probabilistic framework for the analysis of single‐cell RNA sequencing data. scVI takes as input a cells × genes matrix of transcript counts *x* and utilizes the VAE framework to infer the underlying data distribution. The model is generative in that it learns the conditional distribution *p*(*x*|*z*) where *z* is a low‐dimensional latent representation of the data (cells × *k* matrix, where *k* is usually in the few dozens). The conditional distribution *p*(*x*|*z*) describes the probability to see any given number of transcripts for each gene in each cell and builds off advances in the use of count likelihoods for scRNA‐seq data (Risso *et al*, 2018). This distribution can be used for hypotheses testing, such as differential expression. The procedure also approximates the posterior *p*(*z*|*x*), which provides a representation of each cell in the latent space. These representations capture the most important characteristics of each cell and thus provide an effective way for stratifying cells into biologically meaningful clusters or gradients. The model can be used for a range of additional tasks, such as data denoising and imputation (by generating the full‐dimensional data from the latent space), as well as batch effect correction.

**Figure 2:**
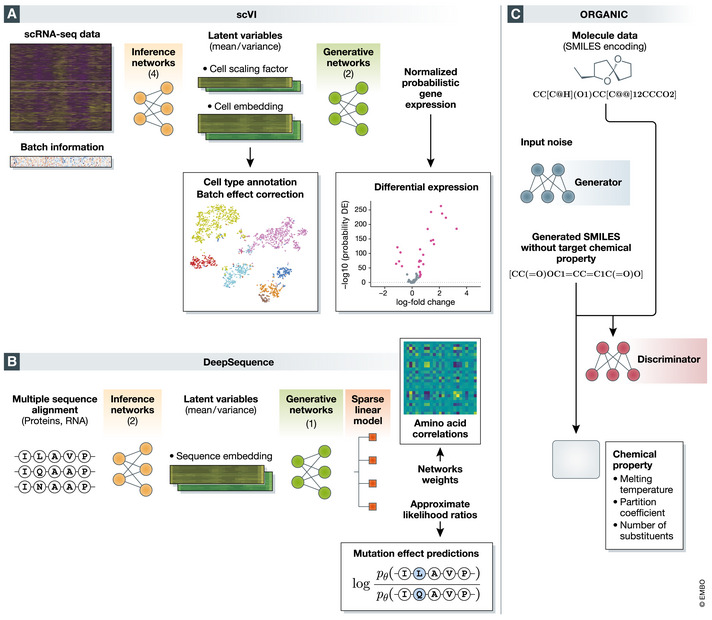
Presentation of the selected models which will be studied in depth for this review (A) scVI, (B) DeepSequence, and (C) ORGANIC

The second model, DeepSequence (Riesselman *et al*, 2018) (Fig 2B), aims to identify deleterious mutations in amino acid or nucleotide sequences with the underlying assumption that functional importance is associated with evolutionary conservation. DeepSequence takes as input sets of homologous sequences *x* from different organisms and fits a VAE to estimate the probability that any given sequence will occur in evolution. The model was designed so as to capture some of the evolutionary constraints that gave rise to the diversity within each set of homologs. It especially emphasizes interactions between elements (amino acids or nucleotides) along the sequence that are jointly associated with increased conservation and may thus indicate functionality. DeepSequence has been applied for predicting whether sequence modifications (e.g., single‐nucleotide mutations) are likely to be chosen by natural selection. Such predictions are used as a proxy for predicting the effect of mutations and understanding which ones are more likely to be deleterious. The estimates from DeepSequence were shown to be more accurate than likelihood ratios from state‐of‐the‐art pairwise and site‐independent models when using deep mutational scan data as ground truth. Furthermore, an especially sound modeling aspect of DeepSequence is to place a group sparsity priors on the weights of the last layer of the generative neural network, which yield interpretable amino acid modules. Notably, the obtained amino acid similarity matrix is shown to be correlated with a well‐known substitution matrix BLOSUM62.

Our third leading example is Objective‐Reinforced GAN for Inverse‐Design Chemistry (ORGANIC (preprint: Guimaraes *et al*, 2018; preprint: Sanchez‐Lengeling *et al*, 2017), Fig 2C), whose goal is to design new chemical compounds with desired properties. ORGANIC takes as input a data set *x* of molecular structures (transformed as a string via the simplified molecular input line entry specification [SMILES] encoding) as well as a black‐box “oracle” capable of predicting *y—*a chemical property of interest (such as fluorescence) for any given molecule. ORGANIC trains a GAN to distinguish between molecules that have the property versus molecules that do not. Notably, it proposes to learn a biased generative distribution so that its output is shifted toward regions in the molecule space that satisfies or maximizes some chemical quantitative properties. Using the trained model, ORGANIC generates novel molecules with a distribution skewed toward high scores with respect to the chemical property of interest *y*. For example, it has been employed to generate drug candidate molecules, by maximizing properties such as melting temperature, chemical beauty, melting point, or Lipinski's Rule of 5. ORGANIC and similar algorithms were shown to generate molecules with significant similarity to existing drugs.

## Brief overview of deep generative models

In the following, we discuss some of the considerations for designing studies with generative models and provide a brief overview on the two primary DGM types, namely the VAE and the GAN.

### Designing generative models

The steps to construct a generative model are best described by Box's loop (Blei, 2014) (Fig 1). This procedure, originally proposed by Box (1976) and Box & Hunter (1962), traverses through the main steps of most applications: choosing a model (e.g., selecting the model type, the input covariates, and the distributions that describe these covariates), inferring parameter values (i.e., training), and criticizing the trained model (e.g., evaluating its accuracy on various tasks). After criticism, we return to the first step and iterate through the loop until we are ready to “deploy” our model.

An important consideration during model design is its *interpretability*—in the sense that its parameters will either be immediately useful (e.g., quantifying interactions between pairs of amino acids (Riesselman *et al*, 2018)) or can be used in downstream analysis to extract knowledge (e.g., cell‐to‐cell similarity in Lopez *et al* (2018a)). It is often the case that there is a clear trade‐off between model complexity and interpretability. Generative models that consist solely of linear operations (such as linear discriminant analysis or factor analysis) are easily interpretable since their parameters normally pertain directly to the input covariates (e.g., one coefficient per gene). This desirable property, however, comes at the cost of a limited ability to fit the data closely. Alternatively, models that use non‐linear operations in neural networks (as it is the case for VAEs and GANs) are normally treated as black boxes whose parameters are not interpretable. These models, however, usually provide a better fit to the observed data and an increased capacity to generalize upon it (e.g., Riesselman *et al* (2018)). The choice of the right trade‐off therefore depends on the prospective uses of the model.

Next, we perform inference over the parameters (i.e., fit the model to data). Since exact inference is usually impossible for most types of generative models, one must rely on approximate inference schemes. In this review, we focus on specific flavors of approximate inference methods that rely on neural networks. Our choice is motivated by a slew of theoretical and engineering advances that makes the task of training generative models significantly more approachable than in the past.

The immediate next step following model fitting is model criticism. This is achieved by defining a set of attributes that we would like our model to have and a set of metrics to evaluate these attributes. A widely used attribute is the capacity to generalize, namely to properly describe data points that were not available during training. Relevant metrics include the likelihood of held‐out data points (Wallach *et al*, 2009) or differences in summary statistics between observed and generated data (posterior predictive checks, [PPC] (Rubin, 1984)). In the latter procedure, we sample random duplicates $\tilde{x}$ of a sample x* (often a group of samples) from the approximate posterior predictive distribution $p\left(\tilde{x}|x^{*}\right)\approx E_{q(z|x^{*})}p\left(\tilde{x}|z\right)$ and compare this to the original data distribution. The comparison can be made by selecting key statistics for the data under scrutiny and testing for changes in those statistics (e.g., comparing coefficient of variation of each gene in single‐cell transcriptomics (Levitin *et al*, 2019)). These metrics are especially suitable for evaluating Bayesian models but may not be defined for other DGMs (e.g., GANs). Another way to approach this is using *a priori* knowledge that is not explicitly available during training, yet should be captured by any useful model. In the context of scVI, one can quantify the extent to which known subpopulations of cells are grouped together in the inferred latent space. In the context of DeepSequence, one can quantify the extent to which the fitted model captures known residue–residue interactions or how well the model predicts deleterious mutations.

### Encoding knowledge with likelihood‐based generative models

A first strategy for generative modeling consists of explicitly defining a likelihood function for the observed data. Our goal is to define a distribution *p*_*θ*_(*x*) from which each observation *x* has been generated, where *θ* denotes the parameters of our model. For instance, in the context of DeepSequence, *x* represents a sequence of amino acids and the likelihood of *x* (parameterized by *θ*) reflects the extent of co‐occurrence in evolution of the amino acids that it contains. The generative model in DeepSequence is a generalization of the so‐called Ising model, which estimates the likelihood that *x* has been selected in evolution by summing over pairs of sites (Hopf *et al*, 2017).

A common Bayesian approach to defining *p*_*θ*_(*x*) is not to model *x* directly, but instead use an unobserved (latent) random variable *z* as an intermediary. That is, to generate a new observation *x*, we first draw an intermediary value *z* from a prior distribution *p*_*θ*_(*z*) and then sample from the conditional distribution *p*_*θ*_(*x*|*z*) (Wainwright & Jordan, 2008). There are two primary reasons for this modeling strategy. First, conditioning on *z* allows us to decouple the contribution of individual entries in *x* (e.g., genes in scVI or positions along a sequence in DeepSequence) to the overall likelihood of *x* (i.e., a cell in scVI or an entire sequence of amino acids in DeepSequence). Since each entry in *x* is assumed to be independent of all other entries when we condition on *z*, the inference of the parameters *θ* becomes substantially simpler. This simplification, for instance, aids DeepSequence to model high‐order dependencies between amino acids, going beyond more traditional models (Hopf *et al*, 2017) that account only for pairwise interactions. Second, in many applications, *z* is of much lower dimension than *x* and therefore provides a concise summary of the data. For instance, in scVI, *z* represents the cell state in a low‐dimensional space (typically set to <20 dimensions) and summarizes the high‐dimensional observations of gene expression (usually thousands of genes). Note that using this property requires a mapping from each observation *x* back to its representation *z* in latent space. We discuss one way to achieve this in the next section.

With the use of intermediate variable *z*, the marginal probability (also termed the *evidence*) of a given data point can be formally written by the a combination of the prior *p*_*θ*_(*z*) and likelihood *p*_*θ*_(*x|z*):

(1) $$\log p_{\theta }\left(x\right)=\log\left(\int \left[\prod_{j=1}^{d}p_{\theta }\left(x^{j}|z\right)\right]p_{\theta }\left(z\right)dz\right),$$

where *d* is the dimension of each observation (e.g., length of sequence in DeepSequence), and *x* ^*j*^ denotes the *j* ^th^ entry of observation *x*. Notably, if the prior is isotropic Gaussian z∼N(0,I) and the conditional likelihood is Gaussian with a linear link $N(u_{j}^{T}z+v_{j},\sigma_{j}^{2})$, then this formulation is a Bayesian version of principal component analysis (as we are specifying a prior for each entry of the principal components), otherwise known as factor analysis (Jolliffe, 1986).

While many practical applications indeed fix the prior to be isotropic Gaussian, the conditional distribution *p*_*θ*_(*x* ^*j*^|*z*) usually comes in other forms that better reflects the nature of the data. For instance, scVI uses the negative binomial distribution—a choice that adequately captures technical (Love *et al*, 2014) and biological (Grün *et al*, 2014) noise in the observed transcript counts. It also includes a possible addition of a zero‐inflation component to further account for sparseness (Clivio *et al*, 2019). Finally, scVI also supports the modeling of the conditional distribution *p*_*θ*_(*x*|*z*,*s*) where *s* denotes the batch information (treated as an observed random variable). This conditional VAE (Louizos *et al*, 2016) setting makes it particularly useful for removing batch effects (preprint: Xu *et al*, 2020). Apart from scVI, DeepSequence uses a categorical distribution to model the occurrence of specific amino acids or nucleotides at each position in the sequence. This distribution also accounts for correlations between different amino acids, thus providing further insight from the model.

### Fitting a generative model using variational inference with neural networks

Once we specified the form of the distributions (prior and conditional likelihood) in our generative model, the inference task is twofold. First, we search a set of parameters *θ* that maximizes the evidence for the data (equation (1)). In parallel, for a complete model we infer the *posterior* distribution *p*_*θ*_(*z*|*x*) that provides a way to represent our observations in the low‐dimensional latent space. While the optimal parameters *θ* can be computed precisely for a restricted choice of distributions (e.g., in factor analysis (Jolliffe, 1986) or other cases where the prior is conjugate to the likelihood), exact inference is intractable for most real‐world applications. Indeed, evaluating the evidence requires integration over the latent variable *z*. Either this quantity does not have a closed form expression, or it may take exponential time to compute (Jordan *et al*, 1999). The same caveat also applies to evaluating the posterior distribution. To see this, recall that Bayes rule entails that:

(2) $$p_{\theta }\left(z|x\right)=\frac{p_{\theta }\left(x|z\right)p_{\theta }\left(z\right)}{p_{\theta }\left(x\right)}.$$

The numerator in this equation can be readily computed in most applications since the prior and likelihood come with a prespecified closed‐form density. However, the denominator is the intractable evidence term.

The main idea behind variational inference is the realization that the problems of maximizing *p*_*θ*_(*x*) and approximating *p*_*θ*_(*z*|*x*) are very much related. As one way to see this, assume that our goal is to find a distribution *q*_*ϕ*_(*z*_*i*_) (also known as the *variational posterior*) that, for a given *θ*, best approximates the posterior. In other words, for every observation *x*_*i*_, its variational posterior *q*_*ϕ*_(*z*_*i*_) should be as similar as possible to the actual posterior *p*_*θ*_(*z*|*x*_*i*_). Because the evidence decomposes across data points, we focus here on the case of a unique observation. Using Bayes rule, we have for each value of *z*:

(3) $$\log p_{\theta }\left(x\right)=\log\frac{p_{\theta }\left(x|z\right)p_{\theta }\left(z\right)}{p_{\theta }\left(z|x\right)}.$$

We can therefore take the expectation of both sides of equation (3)) with respect to *q*_*ϕ*_(*z*) to decompose the evidence as follows:

(4) $$\begin{array}{cc} \log p_{\theta }\left(x\right) & =E_{q_{\phi }\left(z\right)}\log\frac{p_{\theta }\left(x|z\right)p_{\theta }\left(z\right)}{p_{\theta }\left(z|x\right)}\\ & =E_{q_{\phi }\left(z\right)}\log\left(\frac{p_{\theta }\left(x|z\right)p_{\theta }\left(z\right)}{q_{\phi }\left(z\right)}\cdot \frac{q_{\phi }\left(z\right)}{p_{\theta }\left(z|x\right)}\right)\\ & =E_{q(z)}\log\frac{p_{\theta }\left(x|z\right)p_{\theta }\left(z\right)}{q_{\phi }\left(z\right)}+E_{q_{\phi }\left(z\right)}\log\frac{q_{\phi }\left(z\right)}{p_{\theta }\left(z|x\right)}\\ & =\underset{\mathrm{Evidence}\mathrm{lower}\mathrm{bound}(\mathrm{ELBO})}{\underbrace{E_{q_{\phi }\left(z\right)}\frac{p_{\theta }\left(x|z\right)p_{\theta }\left(z\right)}{q_{\phi }\left(z\right)}}}+\underset{\mathrm{Variational}\mathrm{gap}}{\underbrace{\Delta_{\mathrm{KL}}\left(q_{\phi }\left(z\right)\left|\right|p_{\theta }\left(z|x\right)\right)}} \end{array}$$

where ∆_KL_ denotes the Kullback–Leibler (KL) divergence, a notion of similarity between probability distributions. In equation (4)), the variational gap quantifies how well *q*_*ϕ*_(*z*) approximates the posterior and is always positive because that is the KL divergence between two distributions. Consequently, the ELBO is indeed a valid lower bound on the evidence.

Variational inference avoids the intractability of the evidence by maximizing the ELBO, which helps address both of our inference problems. The ELBO includes two sets of parameters. The first set *θ* controls the generative model, and the second set *ϕ* controls the variational approximation to the posterior. Maximizing the ELBO with respect to *ϕ* for a fixed *θ* yields an approximation to the posterior *p*_*θ*_(*z*|*x*), and the ELBO approaches the marginal probability *p*_*θ*_(*x*). In practice, however, we do not have *θ* and the optimization procedure includes assignments of values to both the generative model parameters *θ* and the variational posterior parameters *ϕ* so as to maximize the respective ELBO.

While the ELBO optimization problem is well studied (Blei *et al*, 2017), recent advances in the field provide an effective way to address it using stochastic optimization (Hoffman *et al*, 2013) as well as neural networks, leading to substantial increase in scalability and (for large enough data sets) accuracy. A notable way to achieve this is with VAEs (Box 3) (Kingma & Welling, 2014; Rezende *et al*, 2014). VAEs provide a way for explicitly representing and then jointly inferring both the variational posterior and the generative model. A standard VAE model consists of two components: an *encoder* neural network that maps any given point in the observation space (*x*_*i*_) to its corresponding location in latent space (*z*_*i*_). The mapping is done by setting the parameters of the variational posterior *q*_*ϕ*_(*z*_*i*_) for any given observation *x*_*i*_ through a function *f*_*ϕ*_ represented by the encoder network. In this notation, *ϕ* refers to the weights of the encoder network. For example, with a Gaussian variational approximation *q*_*ϕ*_(*z*_*i*_) = Normal(*μ*_*i*_, diag(*v*_*i*_)), we have (*μ*_*i*_, *v*_*i*_) = *f*_*ϕ*_(*x*_*i*_). Because we can compute *μ*_*i*_ and *v*_*i*_ with a neural network, we do not need to store these values in memory (by opposition to classical variational inference). Notably, because we can obtain the values of the variational parameters at any observation *x* (using the encoder network), we refer to the variational distribution as *q*_*ϕ*_(*z*|*x*). The second component is a *decoder* neural network that maps any given point in the latent space (*z*) to the space of observations (*x*). The mapping is done by setting the parameters of the generative model *p*_*θ*_(*x*|*z*). Notably, neural networks can be particularly useful in cases where linear assumptions might seem inappropriate, which is often the case for biological and medical applications (e.g., statistical relations between genes or loci in a sequence). For instance, in scVI, the mapping from gene expression space *x* to latent space *z* is done non‐linearly by a neural network. The resulting latent representation of cells therefore reflects a notion of cell state that may include complex patterns of gene expression (e.g., a cell state may be defined as the expression of gene A and gene B but not gene C).

At the end of optimization, we have access to a model that fits the data well (in terms of evidence), as well as an approximation of the latent variables’ distribution for each data point. In the example of scVI, the approximate variational posterior over latent variable *z* is used for embedding the cells into the low‐dimensional manifold (e.g., using 10–20 latent dimensions to summarize thousands of genes). Importantly, the distribution *q*_*ϕ*_(*z*|*x*) also measures uncertainty that can be utilized for accounting for measurement uncertainty while performing hypothesis testing (such as differential expression) (Lopez *et al*, 2018a).

**VAEs**

A *variational autoencoder* (VAE) jointly performs learning of parameters for *p*_*θ*_(*x*,*z*) as well as variational inference, with the autoencoder neural network architecture. From the neural network perspective, a VAE contains an encoder network, which maps the observed data points to the distributional parameters of their latent variables (corresponding to the variational posterior *q*_*ϕ*_(*z*|*x*)). It also includes a decoder network, which maps a sample of the latent variables to the parameters of the data likelihood distribution (corresponding to the generative model *p*_*ϕ*_(*x*|*z*)). From the probabilistic perspective, a VAE describes a specific way to perform approximate Bayesian inference. An inference network (encoder) amortizes the cost of inference over a shared set of *variational parameters*,* ϕ*. Meanwhile, the data likelihood distribution is parameterized by neural network (decoder) with parameters *θ*. There are two tasks when training a VAE: (i) learn an approximate posterior distribution over the latent variables, and (ii) update the model parameters such that the data have high likelihood. These two tasks are simultaneously accomplished by optimizing the ELBO with respect to *ϕ* and *θ*.

A rich literature surrounds the VAE, both in applications and in method development. For instance, the VAE has been applied to image generation (Gregor *et al*, 2015), object segmentation with partial observation (Sohn *et al*, 2015), and astronomy (Ravanbakhsh *et al*, 2017). Others have focused on improving parts of the framework such as a different (possibly tighter) lower bound (Burda *et al*, 2016; Li & Turner, 2016), better posterior approximation (Kingma *et al*, 2016), more flexible choices of distributions (Ruiz *et al*, 2016), and richer family of graphical models (Johnson *et al*, 2016).

Figure: Computational Schematics of the VAE.

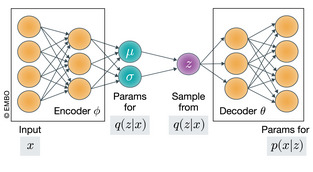

### Likelihood‐free generative models

A competing paradigm for designing generative models is to use *implicit modeling*. Instead of defining a set of conditional probabilities as before, implicit generative models learn to mimic (or simulate) the data by transforming a “pure noise” input channel into outputs that have a similar distribution as the observed data (Sugiyama *et al*, 2012). We refer to these models as likelihood‐free since their fitting procedure does not involve explicit optimization of a likelihood‐related objective, as is the case for the ELBO in VAEs.

Likelihood‐free models can be appealing when we can more readily summarize the knowledge accumulated on some data in the form of a stochastic algorithm (e.g., a simulator or structural equation models) rather than a set of conditional likelihoods. For example, SymSim (Zhang *et al*, 2019) is a simulator for scRNA‐seq data that define an implicit likelihood model by explicitly simulating different processes (biological and experimental) that contribute to the variation in scRNA‐seq (e.g., PCR amplification). Conversely, scVI explicitly defines a set of conditional distributions and is an explicit likelihood model.

A notable example of this family of models is the GAN (Box 4) (Goodfellow *et al*, 2014), in which two competing modules (implemented as neural networks) are jointly optimized. The generator module *G* learns how to transform an input noise distribution *p*(*z*) (e.g., an isotropic Gaussian) into the underlying data distribution *p*_data_ (*x*) (namely, the empirical distribution defined by the space of all observations). The discriminator module *D* learns to distinguish between the empirical data *x* and the data that were artificially generated *G*(*z*). *G* and *D* play a game—namely the two weights in the two networks are optimized in parallel, such that *G* aims to minimize and *D* aims to maximize the following objective:

(5) $$\min_{G}\max_{D}E_{x∼p_{\mathrm{data}}\left(x\right)}\left[\log D(x)\right]+E_{z∼p(z)}\left[\log\left(1-D\circ G(z)\right)\right],$$

where the output of the discriminator network *D*(*x*) ∈ (0,1) estimates the probability that the provided input *x* resembles a true observation. The notation D∘G(z) refers to composition of the two neural networks (i.e., *G* takes *z* as an input, and the output serves as an input for *D*). The optimization of the objective above involves finding an equilibrium instead of a simple extremum. For this reason, a number of contributions focused on stabilizing the training procedure by, for example, changing the objective function (e.g., Wasserstein GANs (Arjovsky *et al*, 2017)).

**GANs**

A *generative adversarial network* places two neural networks in a competitive game. A generator network *G* maps random noise to the space of observations (i.e., trying to generate a “realistic looking” data point). In parallel, a discriminator network *D* aims to estimate the probability that an imputed data point was an actual observation rather than being artificially generated by *G*. The game is structured such that *D*'s goal is to accurately predict the generating source of a data point, while *G*'s goal is to output realistic data points and “trick” *D*. This is formalized in equation (4)).

Figure: Computational Schematics of the GAN.

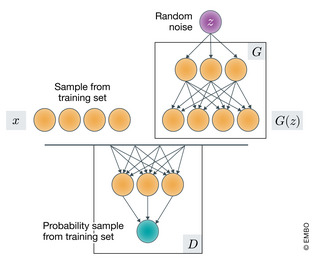

Since their inception in 2014, GANs have been remarkably successful, especially in the domains of robotics (Kurutach *et al*, 2018) and computer vision (Ledig *et al*, 2018). For instance, using large databases of pictures, GANs can be trained to generate new images that are much clearer than those generated by VAEs (Radford *et al*, 2016). This may be attributable to the adversarial loss (equation (4)) that makes GANs more prone to memorizing the training data. Recent work now aims to rectify this, in cases where generalizability is preferable to the production of clear images (Wu *et al*, 2019).

In biological research, GANs have fewer significant contributions compared to VAEs. There are several possible reasons for this. One disadvantage of GANs compared to VAEs is that they do not provide an immediate way of tracing back the representation of each observation in the latent (input noise) space *z*. We note, however, that recent work now aims to address this problem, such as the bidirectional GAN (Donahue *et al*, 2017). Additionally, it may be harder to incorporate our knowledge on the noise structure in the case of GANs, by opposition to VAEs (e.g., enforcing sparsity motifs in the likelihood function *p*(*x*|*z*) for better interpretability of DeepSequence). Consequently, GANs cannot be readily used for dimensionality reduction or for estimating the uncertainty around the observed values. Finally, except in the case of images (at which GANs excel), it may be more difficult to provide clear metrics of how “realistic” a generated data point is. Therefore, it might be harder to justify the use of GANs in many other biological applications.

One notable use of GANs in molecular biology is the ORGANIC method for molecule design (preprint: Sanchez‐Lengeling *et al*, 2017; preprint: Guimaraes *et al*, 2018). The generator network of ORGANIC takes as input random noise (in this case a uniform distribution on the unit hypercube) and maps it to a SMILES encoding of a molecule via a recurrent neural network *G*. The discriminator *D* is a convolutional neural network that takes as input a SMILES encoding and outputs a prediction for whether it represents an observed molecule (available as a part of a training set) or an artificial one (generated by *G*). During the training procedure, the generator network gains the capacity to simulate molecules that are similar to the ones included in the observed training set. To make this property particularly useful, ORGANIC adjusts the standard GAN so as to generate molecules that have certain chemical properties of interest (such as melting point). This is accomplished by adding another part to the objective that rewards the generation of such samples. The balance between the penalty incurred from the discriminator (penalizing unrealistic molecules) and the reinforcement part (penalizing molecules that do not have the desired property) is controlled by a hyperparameter, set before training.

For the sake of completeness, we remark that the boundary between implicit and explicit likelihood models can be blurry. Recent research work shows formal connections between implicit and explicit generative models (Hu *et al*, 2018). For example, some variational inference approaches have been developed for likelihood‐free models (Tran *et al*, 2017b), and conversely, VAEs have been formulated in an adversarial learning context (Mescheder *et al*, 2017). Furthermore, certain types of autoencoders can be trained via adversarial mechanisms such as the adversarial autoencoder (AAE, (Makhzani *et al*, 2016)). Finally, we note that another type of generative model often used in practice is the denoising autoencoder (Vincent *et al*, 2010) (DAE), which can be connected to the VAE (Bengio *et al*, 2013).

## Applications to molecular biology and biomedical research

In the following, we discuss the use of DGMs in various areas of research in molecular biology and biomedicine. We highlight applications to high‐throughput molecular data (e.g., using DNA sequencing to study DNA or RNA, or structural information for proteins) and biomedical imaging. First, we return to our three leading examples and describe how they use DGMs for practical analysis. Then, we group the remaining methods by the tasks they accomplish using DGMs. These tasks include *pattern recognition* procedures (e.g., learning metrics of similarity between data points) as well as *decision‐making* procedures that evaluate and then incorporate uncertainties in the observations (e.g., detecting genes with significantly different expression levels across cell types). We describe how these methods are promising for learning new biology as well as how they may improve over simpler baselines.

### Investigating hidden structure

**Figure 3:**
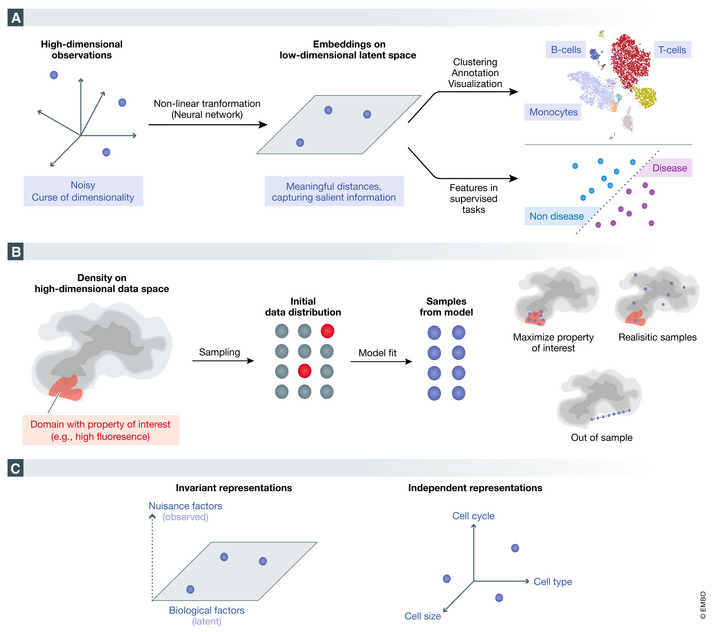
Overview of selected applications of DGMs Learning meaningful low‐dimensional representations (embeddings) from high‐dimensional data to aid both supervised and unsupervised downstream tasks.Generation of novel samples that either (i) satisfy some interesting property, (ii) are mimicking the input data‐generating process, or (iii) are out of sample.Enforcing structure in low‐dimensional representations such as invariance to observed nuisance factors (e.g., batch effects in scRNA‐seq data) or disentanglement. In a disentangled representation, each dimension in a represents variation due to one notable underlying source of variation (e.g., cell cycle).

Biological data are often high‐dimensional. Consequently, statistical models that operate directly on the observed data may suffer from reduced ability to extract clear patterns. This phenomenon may be attributed to the so‐called curse of dimensionality, according to which data points are expected to become more equidistant from each other as the dimension grows (Bellman, 1966). In this context, DGMs provide an attractive framework since they can effectively project the data onto a low‐dimensional space. The resulting *latent representation* or data *embedding* provides a simpler, yet often cleaner perspective of the data, preserving important sources of variation, while excluding artifacts. In the following, we describe how this salient information extracted by DGMs may be useful for both unsupervised and supervised learning tasks (Fig 3A).

#### Embeddings in unsupervised tasks

Applications of DGMs to scRNA‐seq data emerged as a useful way to embed and analyze cells in a low‐dimensional space that summarizes their transcriptomes. Here, the distances between cells in the embedding space can be used to identify phenotypically coherent groups of cells, reflecting either discrete cell types (e.g., T cells, B cells), hierarchies of types (e.g., subtypes of T cells), or variation along some continuum (e.g., progression along the cell cycle) (Ding *et al*, 2018; Lopez *et al*, 2018a; Wang & Gu, 2018; Amodio *et al*, 2019; Eraslan *et al*, 2019b; Rashid *et al*, 2019; Grønbech *et al*, 2020). For example, scvis (Ding *et al*, 2018) employs a VAE to learn a biologically meaningful two‐dimensional representation of single cells from oligodendroglioma samples. Groups of cells that appear as clusters in these two dimensions represent different cell types that are present in the tumor, and characterize its microenvironment. Two‐dimensional projections with scvis were also shown to preserve the structure of high‐dimensional synthetic data better than the popular method t‐SNE (Van Der Maaten & Hinton, 2008) (evaluated by preservation of nearest‐neighbor relationships).

While the layout of cells in the two‐dimensional latent space of oligodendrogliomas reflected the presence of different cell types, the general question of interpreting the meaning of any given low‐dimensional embedding of single‐cell transcriptomes remains challenging. Specific questions include: what does the proximity of cells in latent space mean? Are there areas of the latent space that contain cells with some joint phenotype? And if so, where are these areas and what are these joint phenotypes? One way to approach these questions is relying on gene signatures (DeTomaso *et al*, 2019; preprint: Simon *et al*, 2019) to guide the biological interpretation of any given latent space. These methods first characterize cells based on the expression of gene signatures (e.g., expression changes indicative of stimulation of a T cell) and then look for local neighborhoods in latent space with a higher or lower than expected values of these signatures (using an autocorrelation statistic). A closely related subject is that of ascribing meaning separately to each component of the embedding, which we discuss in the section Disentangling factors of variation.

In the example of DeepSequence (Riesselman *et al*, 2018), the embedding has a phylogenetically coherent structure. For example, the *β*‐lactamase family is mostly organized by bacterial species (e.g., acidobacteria and actinobacteria). Because this information is included in the deep mutational scans studied in Riesselman *et al* (2018), these embeddings do not provide much supplementary information. However, they indicate that the model indeed learned something biologically meaningful from the data.

With DGMs, it is also possible to control the geometry of the latent space (two‐dimensional in the case of scvis) through the form of the prior distribution *p*(*z*). Consequently, different choices of the prior could in principle yield different visualizations. In scvis, *p*(*z*) is set to isotropic Gaussian, while in Grønbech *et al* (2020); preprint: Xu *et al* (2020), the prior is a mixture of Gaussians in order to explicitly model discrete cell types. Beyond those simple yet practical distributions, more recent work (preprint: Ding & Regev, 2019) used probability distributions on hyperspheres and hyperbolic spaces as the prior to provide a more efficient and interpretable cell representation. In the future, Bayesian non‐parametric priors, such as the time‐marginalized coalescent (Vikram *et al*, 2019), may be of interest to analyze single‐cell dynamic lineage tracing data (McKenna & Gagnon, 2019).

#### Embeddings in supervised tasks

Embeddings may additionally be used in supervised tasks (e.g., for predicting the drug response of a patient's tumor based on gene expression data). A natural approach is to use some form of regression model trained on the raw data, after adequate normalization. Alternatively, one can work on a low‐dimensional representation of the data, thus avoiding the curse of dimensionality.

This approach was taken by the authors of DeepProfile (Dincer *et al*, 2018) who used a VAE to predict response to treatment among patients with acute myeloid leukemia. Here, gene expression levels were measured, transcriptome‐wide, for each of thousands of patients. These high‐dimensional gene expression vectors *x* were then mapped to an 8‐dimensional latent variable *z* using the estimated variational posterior *q*_*ϕ*_(*z*|*x*). The embeddings *z* of patients not included during model training were then used as covariates in a penalized regression framework that predicted response to 160 types of chemotherapies. The use of DeepProfile embeddings led to higher accuracy of prediction, compared to regression with principal components, regression with the raw expression data, or *k*‐means.

The efficiency of this approach may depend on the difficulty of the problem at hand. For example, non‐linear embedding‐based classifiers were shown to be outperformed by simpler regression schemes for predicting broad cell types in scRNA‐seq (preprint: Köhler *et al*, 2019). However, it is expected that embeddings are more powerful than classical methods in more complex classification scenarios.

### *In silico* generation of biomedical data

Generation of biological data often requires substantial resources in terms of both labor and cost. The ability of DGMs to mimic the data‐generating process and simulate novel out‐of‐sample data has yielded promising results, especially in the field of computer vision (Ledig *et al*, 2018). This has subsequently led to applications to biomedical data. For example, we may want to generate protein sequences that maximize some desired property (e.g., fluorescence). We may also want to augment our data set with more (artificial) observations to improve statistical power of other downstream methods, like differential expression. In this section, we cover various applications of DGMs to generating new data (Fig 3B).

#### Simulating from data‐generating distributions to enhance performance of downstream analysis

One application of simulating new data is to generate, for each data point, a set of similar pseudo‐observations, thus artificially inflating the number of observations, which can be useful for a range of applications (Shrivastava *et al*, 2017; preprint: Ghahramani *et al*, 2018; Marouf *et al*, 2020). A closely related notion dates to early applications of deep learning in computer vision, where a common practice was to feed neural networks with the same image, shifted and rotated several times. This practice artificially increases the size of the data set and ensures that neural networks can effectively learn properties of the data that are invariant to nuisance factors such as the angle of the picture. For instance, for the now classical task of detecting the presence of a cat in an image, this augmentation was helpful since the rotation of an image does not alter the presence of a cat in this same image.

A recent application of data augmentation in biology was suggested for analyzing scRNA‐seq data (preprint: Ghahramani *et al*, 2018; Marouf *et al*, 2020). For example, Marouf *et al* (2020) downsampled a particular cluster of 15,000 cells in a data set of 68 thousand peripheral blood mononuclear cells. They selected only 0.5% of this cluster so as to simulate a rare subpopulation. In this setting, Marouf *et al* use a GAN to learn the conditional distribution *p*_*θ*_(*x*|*c*) where *c* denotes the cell type information. Such a model is called a conditional GAN (preprint: Mirza & Osindero, 2014). Once trained, the conditional GAN may simulate a large amount of artificial cells that augments the subsampled data set. The authors then performed rare cell type classification on the augmented data using a random forest classifier. When compared to naive upsampling methods, the GAN upsampling procedure yields higher accuracy on a test set. Consequently, they demonstrated that augmentation increased their ability to identify rare cell types. It is reasonable to assume that this type of data augmentation may also improve other downstream scRNA‐seq tasks like differential expression or clustering.

Generative models have also been successful at simulating images, either from electronic microscopy (Han *et al*, 2018b), fluorescence microscopy (Goldsborough *et al*, 2017; Osokin *et al*, 2017; Lafarge *et al*, 2019), or brain magnetic resonance (Han *et al*, 2018a). Lafarge *et al* (2019) developed VAE+, a deep generative model for fluorescence microscopy cell images based on a VAE learned with an adversarial mechanism. The proposed model shows competitive generative capacities and highly discriminative power for classifying compounds. More importantly, VAE+ generates realistic cell images from and in between different treatment conditions, which makes it a particularly powerful tool to visualize how a compound affects cellular structure. In the case of magnetic resonance imaging, Han *et al* (2018a) proposed a GAN architecture that simulates realistic brain images (as measured by the visual Turing test) with the goal of improving the training of image segmentation models as large training data sets can be expensive to obtain.

Finally, instead of simply simulating more data from the same distribution, a sometimes more useful application is super‐resolution, that is the simulation of higher resolution data. For example, Chen *et al* (2018) used a conditional GAN to learn the relationship between low‐resolution Hi‐C data and high‐resolution Hi‐C data. In this setting, both low‐resolution and high‐resolution samples are available in finite quantity. However, acquiring high‐resolution Hi‐C data is often too costly experimentally because increasing the resolution by a linear factor requires a quadratic increase in the number of reads (Chen *et al*, 2018). Therefore, the authors sought to approximate high‐resolution data from low‐resolution data in silico with a GAN. The GAN framework of Chen *et al* also generalizes well across domains, meaning that it can be trained on one cell type and applied to others with high accuracy.

Generation of synthetic observations can improve downstream analysis of molecular data in many cases, as can be seen in the examples listed above. However, it is important to keep in mind that DGMs require a large amount of data in order to learn an accurate distribution for the data‐generating process (refer to Box 5 for further details about this practical concern). Additionally, the learned model might be biased, even with large amounts of data. A solution to alleviate this concern consists of correcting the model's distribution based on importance weighting (Grover *et al*, 2019). This may be useful for more systematic use of generative models for synthetic data generation.

**Number of data points**

One practical consideration to determine whether a problem is amenable to deep generative modeling is whether we have a sufficient amount of observations to learn such a model. What matters here is not only the number of data points but also the complexity of the model (e.g., number of parameters) as well as the complexity of the data (e.g., dimension for the input data). Naturally, as dimensionality and model complexity increase, so does the amount of data needed to fit a model that is robust (i.e., does not change much in response to small perturbations in the data or training procedure) and that generalizes well (i.e., explains unobserved data well). This may explain why most applications use a low‐dimensional embedding (between 10 and 32 for our selected methods), in order to keep a small model complexity. However, latent spaces of higher dimension might help learn disentangled representations as well as provide more expressive models (Mairal *et al*, 2009) in the future.

Most of the frameworks presented in this study have indeed been applied to large data sets. For instance, scVI has been tested with data sets of thousands and up to a million single‐cell transcriptomes. DeepSequence was applied to several data sets derived from multiple sequence alignments, one of which is approximately 100,000 sequences of amino acids in the order of length 100. Nevertheless, when data availability is an issue one can attempt to decrease the required amount using heuristics such as *a priori* limiting the dimension of the data (e.g., using feature selection as in Lopez *et al*, (2018a)) or limiting the complexity of the model. For example, DeepSequence utilized the group sparsity prior to encourage most locus–locus interaction parameters to have a zero value, thus effectively reducing complexity and improving generalization.

#### Generating samples that satisfy or maximize a property of interest

A more complex scenario of generating data *in silico* is to simulate samples that satisfy a given set of constraints or that maximize an objective of interest, such as specific chemical properties of candidate drug molecules. The latter problem has been approached in a number of ways. First, CbAS (Brookes *et al*, 2019) uses a VAE to generate molecules conditioned on a constraint on the property of interest (e.g., proteins with a fluorescence greater than a threshold). More precisely, *S* denotes the property of interest (e.g., a certain range of fluorescence), and the objective is to sample molecules *x* from the conditional distribution *p*(*x*|*S*) (approximated with the VAE). The main difference between CbAS and a simple VAE modeling the conditional distribution *p*(*x*|*S*) is that the event *S* can be extremely rare since not many proteins from the training set will have high fluorescence. For this reason, CbAS develops a specific iterative method for generating those “rare” molecules.

As an alternative to sampling from a distribution that is conditioned on a property of interest, one can try to generate samples that maximize it. For example, ReLeaSE (Popova *et al*, 2018) enforces a recurrent neural network to sample molecules that maximize inhibitory activity against Janus protein kinase 2. ORGANIC utilizes a GAN that is penalized for simulating molecules with low values of chemical properties of interest. Overall, such methods might be critical in future developments in drug discovery. For example, in Kadurin *et al* (2017a,b), an adversarial autoencoder (AAE) is trained on a collection of chemical compounds which includes, for every compound, its molecular fingerprint and the response (growth inhibition) of a cancer cell line to treatment at various concentrations. The AAE generates molecular fingerprints with high response with the AAE, which can then be employed for screening a repository of 72 million compounds, for the majority of which the cancer response assay was not conducted. Using molecular fingerprints, the model identified new molecules that can have anticancer capacity. As evidence for the accuracy of this approach, the authors demonstrated that the method was able to re‐discover known anticancer agents that were not included in the training set. For further details of this and other aspects of molecule design, we refer to the review by Sanchez‐Lengeling and Aspuru‐Guzik (2018).

The concept of guided data generation is closely related to the areas of sequential decision‐making, active learning, and reinforcement learning (RL) (Peters & Schaal, 2007). The respective procedures generally include an agent and an environment, in which the agent takes actions based on a limited amount of data and proceeds iteratively to test the property of interest in new samples that it generates. The success of RL applications in molecular discovery therefore largely depends on the ability to evaluate the desired chemical specification in an interactive manner (Mnih *et al*, 2015). In molecule design, a black‐box *oracle* predicts the particular chemical property of a given input molecule (either using molecular dynamics simulations or learned from data). As such, the quality of the oracle is also of great importance to the success of these applications (Schneider *et al*, 2020).

Significant bottlenecks that limit the applicability of RL in molecular biology compared to robotics include lower experimental throughput and higher turnaround time (e.g., due to the need for recruiting donors, preparing samples, and preprocessing data). As the efficiency of experimental platforms (e.g., from sequencing to analysis of higher order chemical properties) is increasing, we believe it is reasonable to expect true RL applications in molecular biology, which will automatically analyze experimental results and (for instance) explore new drug candidates in an online fashion.

#### Generating out‐of‐sample data

Generated data may also extrapolate beyond our observations—namely generating and then drawing conclusions from new data points that are fundamentally different from the observed ones. This can be done by sampling from areas of the latent space to which none of the observed data points is mapped, but that may still have a meaning. A popular way to do that is using the so‐called latent space arithmetic, namely performing linear operations on the latent representation of observed points and generating data from the resulting coordinates. For instance, one may sample points in latent space along a line between two observed data points, presumably spanning all intermediate states between these points. While latent space arithmetic does not have any theoretical guarantees, it has been successfully applied in computer vision, for instance to linearly interpolate between *z*_0_, the latent representation of an image of faces looking left, and *z*_1_, that of a face looking right, thus generating an artificial, yet smooth left‐to‐right transition *x*_t_ (Radford *et al*, 2016).

(6) $$\begin{array}{cc} z_{t} & =tz_{0}+\left(1-t\right)z_{1}\\ x_{t} & =E_{p(x|z_{t})}\left[x\right] \end{array}.$$

An application of this idea in molecular biology was presented by preprint: Ghahramani *et al* (2018) who used latent space arithmetic to simulate epidermal differentiation. Here, a GAN was trained on scRNA‐seq data sets of epidermal cells and a differentiation gradient was estimated in the latent space by subtracting the latent representations of differentiated and undifferentiated cells. This gradient was then used to study epidermal cell differentiation *in silico*. Notably, mapping an observation *x* (here a cell's transcriptome) back to its latent representation *z* is not trivial with GAN since, unlike VAES, they do not include a posterior distribution *p*(*z*|*x*). To resolve this, the authors proposed to randomly sample points from the latent space (i.e., inputs to the generator network *G*) until a point *z*′ is found whose generated transcriptome *G*(*z*′) is sufficiently similar to the desired *x*.

By starting from the latent representation of a specific undifferentiated cell and following the differentiation gradient, new and unobserved points were then sampled at various points along the differentiated‐undifferentiated segment. These points in latent space were then converted to full‐dimensional points (in gene expression space) using the GAN generator. The authors used these simulated profiles to understand the dynamics of the differentiation process and verified that known differentiation markers such as Ppl and Grhl3 increase over the simulated process.

Latent space arithmetic is also central to the method scGen (Lotfollahi *et al*, 2019), which uses a VAE framework to predict the effect of any given perturbation (e.g., chemical treatments) on the transcriptome. First, the authors derive a perturbation vector that represents the difference in latent space between the average transcriptomes of perturbed *x*_p_ and unperturbed *x*_u_ cells. Applying this vector to the latent representation of cells $x_{u}^{*}$ of a different type for which perturbation data are not available results in the generation of perturbed expression profiles ${\tilde{x}}_{p}$, using the formula

(7) $$\begin{array}{cc} \delta & =E_{q(z_{p}|x_{p})}\left[z_{p}\right]-E_{q(z_{u}|x_{u})}\left[z_{u}\right]\\ {\tilde{z}}_{p} & =E_{q(z_{u}|x_{u}^{*})}\left[z_{u}\right]+\delta \\ {\tilde{x}}_{p} & =E_{p(x_{p}|{\tilde{z}}_{p})}\left[x_{p}\right] \end{array}.$$

This generated expression profile is then validated using held‐out data. The authors also showed that this method corrects batch effects (estimated by a gradient representing the latent space difference between batches). We stress that these methods do not come with theoretical guarantees and that conclusions drawn from out‐of‐sample generation require manual inspection. Indeed, DGMs often fail to capture the full extent of the semantics contained in the data set. For example, a data set of shape drawings is used to train a DGM in Zhao *et al* (2018). While each image of the training set contains exactly six colored dots, the images generated from the DGM can contain a variable number of dots. This shows that the model learned only a limited semantic and in a biological setting could generate artifacts that are irrelevant to scientists. This risk can be mitigated by careful assumptions as well as experimental validation.

#### Imputing missing data

Often biomedical data are derived from experimental procedures with low sensitivity or limited scope, resulting in many missing entries in the observed data. For example, most imaging‐based protocols for spatial transcriptomics of single cells (e.g., smFISH (Codeluppi *et al*, 2018)) record for each cell its position in a tissue, while measuring the expression of a certain panel of genes (instead of transcriptome‐wide). In this case, we wish to query the expression for unseen genes to study the spatial determinants of their expression. To address this, our group developed a method that leverages any available scRNA‐seq data (which is genome‐wide, but with no spatial information) of the same biological system (Lopez *et al*, 2019). In the model, cells from both assays (scRNA‐seq and spatial transcriptomics) are embedded in the same latent space using a coupled decoder network that was designed to control for protocol‐specific artifacts. From that latent space, the model generates either type of observation by learning two different decoder networks (one per protocol). This framework has been evaluated using held‐out data, demonstrating that cells profiled with spatial transcriptomics can be “encoded” into a joint latent space with cells profiled with scRNA‐seq and then “decoded” to their predicted full transcriptome.

In a different setup, we are interested in transcriptome‐wide gene expression data in our set of samples, but we only have access to the expression of a small set of landmark genes. An example for this scenario is the Connectivity Map project (Subramanian *et al*, 2017) where expression levels were only measured for a panel of approximately 1,000 genes using a custom‐designed microarray. This made gene expression measurements cost‐efficient and feasible for a very large number of samples. Dizaji *et al* (2018) and Wang *et al* (2018) proposed GAN frameworks to predict (impute) the expression values of the unmeasured genes. Wang *et al* (2018) used a conditional GAN, meaning that the generator takes the landmark gene expression as input and outputs the target gene expression. This approach leverages correlations between the set of landmark and target genes in expression data from projects like 1000 Genomes. Its performance also improves upon baseline linear regression and deep learning models.

Another problem that has received much attention is imputation of scRNA‐seq dropout events (Codeluppi *et al*, 2018; Lopez *et al*, 2018a; preprint: Qiu *et al*, 2018; Deng *et al*, 2019; Eraslan *et al*, 2019b). Dropout is regarded as a technical artifact in which a certain transcript is not observed in a cell despite being present. While some biases may affect dropout rates, it is largely ascribed (especially in the current state‐of‐the‐art protocols) to limited sensitivity (only capturing a small percentage of the transcript in a cell) (Lopez *et al*, 2018a). The imputation of dropped out transcripts has been formulated either as a missing value problem (preprint: Qiu *et al*, 2018; Deng *et al*, 2019) or as querying the mean of unobserved latent variables (Lopez *et al*, 2018a; Eraslan *et al*, 2019b). In the latter approach, one can generate a new count matrix in which dropouts have been imputed and then use this matrix for downstream analysis. For example, Eraslan *et al* (2019b) used such an imputed matrix to estimate a gene–gene covariance matrix of key regulatory genes in blood differentiation. Using the imputed data (instead of the raw data) led to an increase in anticorrelation between Pu.1 and Gata1, which are well‐known megakaryocyte–erythrocyte progenitor and granulocyte–monocyte progenitor regulators. Despite promising results, this approach should be used with caution as it has been observed that imputation may also induce spurious correlations (Andrews & Hemberg, 2018).

In a more clinical application, Rampášek *et al* (2019) learned a VAE on bulk gene expression data sets from cancer cell lines. A small fraction of the data has paired pretreatment and post‐treatment measurements as well as drug response information. However, most data points only have a subset of the paired measurements. Because they use a fully probabilistic model, the label information can be incorporated directly in the model. Their method outperforms baseline discriminative methods in drug response classification performance.

### Disentangling factors of variation

A common task in analyzing large data sets is isolating and interpreting the different sources that contribute to the observed variation (Fig 3C). A related classical problem in statistics and computer science is the one of *blind signal separation* (Hyvärinen & Oja, 2000). In this setting, one observes a superposition of signals and the aim is to recover independent components of variations. A more relaxed version of this problem is simply to summarize the data into useful statistics (e.g., correlations) so that the practitioner can gain insight about contributions of each bit of information to an abstract model. A model or a procedure that achieves this goal will be referred to as *interpretable*.

#### Signal separation in single‐cell transcriptomics

The characterization of a cell by the transcripts it expresses is reflective of many sources of variation—both biological and technical (Wagner *et al*, 2016). The biological component consists of numerous factors such as the type of the cell, its microenvironment, its progression along differentiation or stimulation processes, the cell cycle phase it is at, variation in DNA (e.g., in T‐ or B‐cell receptors), and more. The technical component is governed by nuisance factors that can be either observed (e.g., sequencing depth or batch identifier) or not (e.g., mRNA capture efficiency per cell). The product of a scRNA‐seq experiment is therefore a superposition of mixed effects representing all these sources. In general, it is infeasible to recover the contributions of all the different sources without explicit design, largely because they can be confounded with each other (Wagner *et al*, 2016). This justifies the choice of some methods in a more coarse approach, considering the gene expression data as a mix of between wanted and unwanted variation (e.g., SCONE (Cole *et al*, 2019), RUV (Risso *et al*, 2014), and ZINB‐WaVE (Risso *et al*, 2018)).

One way to address the disentanglement challenge with DGMs is to treat each component of the latent space as a source of variation and then use additional inspection to ascribe biological meaning to each component. In PCA, such an interpretation is straightforward as the components are orthogonal and represent a linear combination of the observed features (Gaublomme *et al*, 2015). Indeed, in many applications these components reflect critical aspects of the data such as geographical locations in population genetics (Novembre *et al*, 2008) and T‐cell effector function in single‐cell RNA sequencing (Gaublomme *et al*, 2015.). Interpretation can be more challenging in the case of DGMs, where the embeddings are (at least in the standard formulations) non‐linear and not necessarily independent.

One way to increase the interpretability of latent components in DGMs is to reduce their association with observed nuisance factors. In the context of scRNA‐seq, scVI uses conditional independence via a graphical model to enforce statistical independence between the components of the latent space and nuisance factors such as cell‐specific scaling (indicative of sequencing depth), batch effects, and propensity for dropouts (Lopez *et al*, 2018a). This type of modeling was also used to integrate scRNA‐seq data sets from different protocols, by conditioning on protocol identifier (preprint: Xu *et al*, 2020). One can also go beyond conditional independence by explicitly optimizing for weak dependence between the latent space *z* and any observed nuisance factor *s*. In principle, this can be done by adding the respective independence term **I**(*z*,*s*) to the evidence lower bound (equation (4)) that we usually optimize for during training

(8) $$\tilde{L}\left(x\right)=\text{ELBO}\left(x\right)+\kappa E_{q(z|x)}I\left(z,s\right)$$

where *κ* is a parameter which governs the trade‐off between the flexibility of the model and the independence constraints. For example, Higgins *et al* (2017) use as an independence term the Kullback–Leibler divergence between the posterior and the prior distribution Δ_KL_(*q*_*ϕ*_(*z*|*x*) || *p*_*θ*_(*z*)). This choice of independent terms forces the VAE to use less bits of information to encode the same image and therefore disentangle semantics of the images (such as shapes and angles). Lopez *et al* (2018b) used instead a non‐parametric measure of dependence, based on kernel methods and applied it to reduce the effect of quality metrics associated with each cell (e.g., number of reads) on its representation in latent space and consequently on any downstream analysis. It does, however, require tuning of the regularization strength *κ*, which may be hard in biological setting because some nuisance variables are confounded with the biology (Vallejos *et al*, 2017).

Up to now, the presented methods learned a generative model conditioned on experimental information *s*. This is not the unique possibility, since other methods in the single‐cell field are still able to isolate variation due to batch effects or protocol‐specific effects. For example, scGen corrects scRNA‐seq batch effects through latent space arithmetic, namely through a subtraction operation in latent space (Lotfollahi *et al*, 2019). Another related method, MAGAN, was developed to merge data from different modalities such as CyTOF (which estimates the abundance of a small panel of proteins in single cells using mass spectrometry) and scRNA‐seq (Amodio & Krishnaswamy, 2018). MAGAN has a different architecture from the classical GAN. In MAGAN, one generator *G*_12_ takes a sample from modality 1 and maps it to values in modality 2. Similarly, a second generator *G*_21_ operates the transformation in the inverse direction. In one of its key innovations over earlier work in computer vision (Zhu *et al*, 2017), MAGAN exploits biological knowledge about feature correspondence (e.g., mapping proteins to their coding gene) in order to make the problem more tractable.

We may also want to understand the variation in one target data set relative to some background data set. For example, if we have skin images collected from a diverse population, our standard methods might pick up on variation due to skin color, age, and gender instead of salient information like skin lesions (preprint: Abid & Zou, 2019). Contrastive learning is a framework that aims to highlight the differences between a background and a target data set with a latent variable model (Abid *et al*, 2018). This framework was extended to use VAEs in preprint: Abid & Zou (2019) with results showing the ability to better categorize blood cells pretreatment and post‐treatment from a leukemia patient when using healthy cells as a background. We note that choosing an appropriate background data set is non‐trivial and will ultimately influence performance.

Finally, we anticipate that a future step for single‐molecule fluorescent *in situ* hybridization (smFISH) data analysis will require to disentangle between variation in gene expression that depends on the position of the cell in the tissue (spatial component) and other biological components that are independent of that position. Gaussian processes are particularly suitable for these tasks, while VAEs are particularly useful for coupling a latent space with count observation. We therefore anticipate a mixture of the two techniques will yield valuable insight to biology scientists on smFISH data (Casale *et al*, 2018).

In this section, we have focused on decomposing embeddings into sets of *uncorrelated* features in order to possibly learn statistical associations between biological phenomena. This approach contrasts with the field of *causal* inference, which aims at drawing conclusions between actions and outcomes, as we further discuss in Box 6.

**Beyond statistical associations and toward causal inference**

Causal inference (Pearl, 2009) provides a principled way of reasoning about actions and outcomes. While generally, causal inference is out of the scope of this review, we will briefly discuss how this paradigm, combined with DGMs, might be applied to questions in molecular biology.

A key class of problems in causal inference relates to counterfactuals. This is an especially common topic in health care, where key questions are of the form “what would have been the treatment leading to the optimal outcome for this particular patient?” Notably, deep generative models have been used to study causal effects (Pearl, 2009) on semi‐simulated data, for marketing or healthcare applications (e.g., individualized treatment effect estimation (Louizos *et al*, 2017; Yoon *et al*, 2018)). However, accurately recovering treatment effect from observational (rather than interventional) data requires first that there are no hidden confounders that control treatment outcome and second that it was feasible to assign to each observed case actions different from the one observed.

Classically, integration of single‐cell transcriptomics data is a critical field of research but does not satisfy the hypothesis mentioned above. For example, let us consider a clinical study performed via scRNA‐seq and in which there are *n* patients for control and *n* others for the phenotype of interest. scRNA‐seq is performed via the same protocol but on different days. Of course, there are probably some hidden confounders such as age, sex, or lineage, but more importantly, each cell is assigned to a single batch since scRNA‐seq is a destructive method. Consequently, having a general understanding of how individual cells react to phenotypical changes is in general intractable and one might resort to more structured hypotheses. For example, scGen (Lotfollahi *et al*, 2019) assumes that in its latent space all cell types between conditions are affected by the same vector translation. While this might be a reasonable assumption in the particular case studied in the manuscript, such an hypothesis may not hold in the more general setting of cell type‐specific response to a stimulation. In particular, such an approach might yield spurious discoveries. Consequently, it may be more suitable to re‐think the experimental design. Indeed, another workaround to identify causal effects is to perform interventions (e.g., force key variables to have some fixed values). A particularly promising framework is Perturb‐seq (Dixit *et al*, 2016), for which CRISPR perturbations (e.g., interventions) make possible the estimation of causal effect for transcription factors or gene modules.

#### Interpretability of deep generative models

Generally, it is difficult to interpret internal hidden neurons of the neural networks of a deep generative model. Still, there are several examples where manual examination or algorithmic procedures can unravel interesting information. Some of the topics we refer to here are related to the field of explainable machine learning (Gilpin *et al*, 2018).

First, it is known that linearity is an efficient way to get interpretability (as in principal component analysis). Therefore, it is quite natural to investigate the last layer of the neural network (which is followed by the output layer) since these neurons are often linked to the output with a linear function. This last linear transformation can be used to discover feature‐specific information either in generative networks of VAEs or in generators of GANs. In preprint: Ghahramani *et al* (2018), a GAN is trained on scRNA‐seq data and a gene–gene covariance matrix is constructed through representing each gene by the weights of the final neural network layer. While this approach is interesting, we note that it comes with no theoretical guarantees and may produce spurious correlations. In Svensson *et al* (2020), a VAE with a linear decoder (LDVAE) is proposed for scRNA‐seq data. The LDVAE trades model fit for more interpretability since the decoder now relates directly latent variables to the expression of individual genes.

For some type of data, features can have a spatio‐temporal structure such as pixels in images, sampling of a time‐series, or sequence data. In this case, a common practice is to structure the architecture in a way that each hidden variable can focus on only a certain region (e.g., area of the image) at a time. These so‐called attention mechanisms (Bahdanau *et al*, 2015) may be an attractive solution for interpreting predictions of a neural network. In Manica *et al* (2019), the multimodal convolutional neural network has an attention mechanism to encode the SMILES string of the chemical compounds as well as gene expression and predict drug sensitivity. Their results suggest that the attention mechanism focuses on relevant genes and functional groups for understanding progression of leukemia. While attention mechanisms have proven to be successful in areas of natural language processing (Bahdanau *et al*, 2015), applications to biology should control at least a measure of risk such as the false discovery rate. Current approaches have no methods for providing such guarantees.

In a certain number of real‐world instances, using a linear model might result in an important loss of performance and the input data might not have spatial structure such as molecular information. In that setting, the Shapley value (the expected marginal contribution over all possible subsets) can be a valuable tool to assess feature importance and interpret models. Although the original Shapley value is computationally intensive, recent research work provided tractable approximations (Lundberg & Lee, 2017) to interpret complex models and large data sets. Such approach has been applied to explainable predictions of anticancer drug synergy (Janizek *et al*, 2019). In this work, Janizek *et al* provide a model which outperforms state‐of‐the‐art predictions and provides biological insight into relevant pathways for understanding drug synergy in leukemia treatment. More systematic use of such tools may be an important direction to take for applications of machine learning in computational biology.

Additionally, a more Bayesian procedure for interpretability is to treat the weights of the neural networks as hidden random variables with a sparsity‐inducing prior (e.g., a Laplacian, Gamma, or a spike‐and‐slab prior), as in DeepSequence. Similarly, the concrete autoencoder (Balın *et al*, 2019) performs combinatorial optimization to find the optimal subset of features which better recapitulates the input data. This latter technique could be applied more widely to investigate feature importance for different hidden random variables for example.

### Utilizing uncertainty for Bayesian decision‐making

The Bayesian model in equation (1)) provides a joint probability distribution *p*_*θ*_(*x*,*y*) that, along with Bayes rule, fully describes the latent variables associated with each data point (i.e., the posterior distribution *p*_*θ*_(*z*|*x*)). Variational methods provide an approximation to this posterior (i.e., *q*_*ϕ*_(*z*|*x*)), and therefore, as soon as the quality of the approximation is sufficient, one can obtain a myriad of uncertainty measures about our observations, such as a credible interval (or region) around the observed value *x*. Estimating and utilizing this uncertainty received much attention in the machine learning literature, especially in the context of active learning and reinforcement learning (Osband *et al*, 2018). More generally, the posterior can be used for any type of Bayesian decision‐making procedure such as hypothesis testing, issuing natural hazard warnings in public policy (Economou *et al*, 2016), and novelty detection in robotics (Amini *et al*, 2018). In this section, we present work that makes implicit appeal to Bayesian decision theory (Berger, 2013) in order to ground scientific discoveries.

One natural utilization of the approximate posterior is to approximate the marginal log‐likelihood of each data point, which can help highlight observations that are not described well by the model. In the context of scRNA‐seq, this concept was used by scvis (Ding *et al*, 2018) to identify novel subpopulations of cells. Specifically, the authors first trained a VAE on a set of mouse retinal bipolar cells. Then, another data set of cells from the entire retina was embedded in the same latent space, without re‐fitting the model. The authors found that non‐bipolar cells from the full retina data set had lower likelihood than bipolar cells. While this trend was expected given our a priori knowledge on the assayed cells, it served to validate this use of VAEs as a general way of identifying subpopulations of cells that are present in a new sample and were not in any previous ones.

A more complex application of uncertainty in molecular biology pertains to decision‐making via likelihood ratios. In the case of scVI, the posterior distribution over the latent space can be used to derive credible intervals of gene expression levels (reflecting measurement uncertainty), thus enabling estimation of differential expression. Indeed, differential expression can be formulated as a Bayesian decision‐making problem (whether the expression of a gene is significantly changing between two populations of cells). Building on this idea, scVI uses a likelihood ratio (comparing the two models of either “differential” or “no difference” in gene expression), approximated by sampling from the posterior distribution *q*_*ϕ*_(*z*|*x*) and then the generative model *p*_*θ*_(*x*|*z*), of cells in the two populations of interest.

Estimation of uncertainty can be used for other critical tasks in scRNA‐seq analysis, beyond differential expression. One example is assignment of cell type labels, which is often done by drawing arbitrary cutoffs between clusters (i.e., with no regards to uncertainty). One way to address this was implemented in scANVI (preprint: Xu *et al*, 2020) where cell type annotations *c* are treated in a semi‐supervised learning fashion (e.g., only labels with high confidence such as from cells expressing key marker genes from a curated gene set). The model learns a likelihood distribution *p*(*x*|*z*,*c*) that conditions the gene expression values *x* not only on the latent space *z* but also on the annotation *c*. This model treats cell types *c* as a random variable and defines a posterior *p*(*c*|*x*) that can then be estimated with variational inference (Kingma *et al*, 2014). This enabled us to provide predictions as well as uncertainty for cell types based on the variational posterior *q*_*ϕ*_(*c*|*x*). Results suggest that this uncertainty can also be useful for differential expression, since we longer compare two fixed sets of cells (which might include misannotated cells), but rather draw from the posterior of these sets.

Another application of uncertainty to decision‐making is the prediction of effects of mutations in DeepSequence. In this work, the main assumption is that the training data are enriched for biological sequences (e.g., proteins) that were selected during evolution and are therefore likely functional. In this setting, the model is assumed to attribute high marginal likelihood to sequences that represent functional proteins (possibly harboring non‐deleterious mutations) and low marginal likelihood to sequences that include deleterious mutations (which were presumably under‐represented in the training set). Interestingly, the likelihood ratio wild‐type *p*_*θ*_(*x*_wt_) to mutation *p*_*θ*_(*x*_m_) is informative of how much evidence there is that mutation *x*_*m*_ is functional or deleterious.

(9) $$\log\frac{p_{\theta }\left(x_{\mathrm{wt}}\right)}{p_{\theta }\left(x_{m}\right)}.$$

These preliminary applications of decision theory to real biological problems are promising and can potentially be applied to more critical problems such as online exploration of treatments or even experimental design. However, these will probably require further developments of the inference mechanisms. Indeed, correctly estimating log‐likelihood ratios or marginal log‐likelihood requires to have at disposal a variational distribution close to the model's posterior. In practice, variational inference does not provide a provably efficient approximation method, and in certain instances, estimations of log‐likelihood ratios can be especially inaccurate. Recent developments in variational inference provide empirical procedures to assess the quality of the approximate posterior distribution (Yao *et al*, 2018), as well as alternative training procedures that yield more suitable posterior distributions (Le *et al*, 2019; preprint: Lopez *et al*, 2020). Another important area for improvement of the presented applications is the control of error (especially of the false discovery rate, [FDR] (Benjamini & Hochberg, 2016)). While this is vastly underexplored in current DGMs applications, the FDR can be in principle controlled using posterior uncertainty (e.g., in RNA‐seq data (Cui *et al*, 2015)). Taken together, we expect that these tools will play an important role in building meaningful and principled applications of VAEs to molecular biology.

## Perspectives

Deep generative models bring to the table both remarkably flexible modeling capabilities and convenient inference procedures. These advantages in practice resulted in promising range of applications in molecular biology. We have highlighted a number of success stories in the field of genomics, single‐cell transcriptomics, molecular design, and more. We also mentioned a few significant areas for improvement such as interpretability of the models, looking for causal relationships, and diagnosing the quality of the inference procedures.

With the recent proliferation of DGMs, more consideration needs to be given to *model selection*: the task of comparing the performance of two or more candidate models. Aspects of model selection that may be of interest include different choices for the family of conditional distribution for the model (as in scVAE (Grønbech *et al*, 2020)) or for the hyperparameters (e.g., neural network architecture or parameters of the optimization procedure; refer to Eraslan *et al* (2019a) for further details about hyperparameters). In all of these scenarios, the methodology developed for *model criticism* applies and should be used whenever appropriate. The starting point is the assessment of the goodness of fit, often done via estimation of the likelihood of held‐out data and PPCs in the case of VAEs. However, recent work showed that held‐out log‐likelihood might not be correlated with performance for certain downstream analyses where biological plausibility is evaluated (Hu & Greene, 2019) (phenomenon also discussed in the machine learning literature (Theis *et al*, 2016)). Consequently, improvements on goodness of fit may not be convincing enough and generative models must therefore be evaluated, whenever possible, in the context of their prospective use. A notable example of evaluation framework is TAPE (Rao *et al*, 2019), which provides public data sets, evaluation metrics, and non‐trivial training–test–validation splits for assessing algorithms for embedding protein sequences. Such an effort in applications ranging from machine learning to molecular biology will allow fast, reproducible, and scientifically relevant methodology developments.

In this review, we focus on probabilistic models as a conceptually attractive way to mine data. DGMs achieve this through their capacity to generate artificial observations that have similar properties as the truly observed ones. We emphasize that this mimicking, however, is usually limited—in many cases, DGMs can only provide a crude representation of actual data‐generating processes, especially in complex settings encountered in biology. Nevertheless, while the saying “all models are wrong” (Box, 1976) is likely to hold here, it might still be the case that “some are useful”. Indeed, properly trained generative models can provide the analyst with a principled way to model the uncertainty in the observations he or she has, generalize upon these observations and draw conclusions from them.

With the increase in experimental protocols that generate large amounts of data (Efremova & Teichmann, 2020) (such as sequencing, microscopy) and the accumulation of large data repositories (e.g., of medical records), we expect DGMs to find numerous new applications and challenges. For example, the case of clinical trial data is particularly sensitive because of privacy issues: It may be possible to identify the participants from inspection of their data. Generative models may then be used to generate a private copy of the data (Dwork, 2008), shareable with others to explore scientific hypotheses about the clinical trial (such as individual treatment outcome with respect to age, sex, etc.) while preserving participants’ privacy (Beaulieu‐Jones *et al*, 2019). Furthermore, one clear advantage is that DGMs are well suited for a joint analysis of data sets from different sources (e.g., experiments from different laboratories) or different types (e.g., harmonizing protein‐ and mRNA‐based single‐cell profiles (Amodio & Krishnaswamy, 2018)). Indeed, given the flexibility of DGMs (e.g., selecting which distribution to use), one can incorporate prior knowledge on the system in hand in terms of its generative process and in terms of the noise that comes with the observations. Finally, software libraries are available that already implement many of the operations needed by DGMs, while making use of modern computational tools such as stochastic optimization, automatic differentiation, and GPU‐accelerated computing. For these reasons and given the scale and complexity of current data sets (e.g., (Regev *et al*, 2017; Davis *et al*, 2018; Bento *et al*, 2014])), DGMs may become an integral part of the standard analysis toolbox in the life sciences.

## Conflicts of interest

The authors declare that they have no conflict of interest.
